# Neighborhood and Housing Conditions and Risk of Falls

**DOI:** 10.1093/geroni/igaa057.2245

**Published:** 2020-12-16

**Authors:** Haena Lee, Jennifer Ailshire

**Affiliations:** University of Southern California, Los Angeles, California, United States

## Abstract

Physical conditions of living environments can impact risk of falls, however, prior work has focused typically on one domain at a time—either neighborhood or home, capturing limited environmental boundaries of older adults. We extend prior work by considering both neighborhood and home as important residential contexts and examine their impact on the onset of falls over time. Data are drawn from two waves of the Health and Retirement Study (2012 and 2016; N = 2,244). We used interviewers’ report on outdoor and indoor dwelling conditions to assess environmental risk factors. One-third of respondents reported at least one fall years later. The presence of green space such as a park near the housing unit and clutter in the home appears to increase the risk of falls over a four-year period. This finding suggests that falls in old age may be determined by a combination of outdoor and indoor risk factors.

